# Measurements of acoustic radiation force of ultrahigh frequency ultrasonic transducers using model-based approach

**DOI:** 10.1063/5.0044512

**Published:** 2021-05-03

**Authors:** Sangnam Kim, Sunho Moon, Sunghoon Rho, Sangpil Yoon

**Affiliations:** Department of Aerospace and Mechanical Engineering, University of Notre Dame, Notre Dame, Indiana 46556, USA

## Abstract

Even though ultrahigh frequency ultrasonic transducers over 60 MHz have been used for single-cell-level manipulation such as intracellular delivery, acoustic tweezers, and stimulation to investigate cell phenotype and cell mechanics, no techniques have been available to measure the actual acoustic radiation force (ARF) applied to target cells. Therefore, we have developed an approach to measure the ARF of ultrahigh frequency ultrasonic transducers using a theoretical model of the dynamics of a solid sphere in a gelatin phantom. To estimate ARF at the focus of a 130 MHz transducer, we matched measured maximum displacements of a solid sphere with theoretical calculations. We selected appropriate ranges of input voltages and pulse durations for single-cell applications, and the estimated ARF was in the range of tens of *μ*N. To gauge the influence of pulse duration, an impulse of different pulse durations was estimated. Fluorescence resonance energy transfer live cell imaging was demonstrated to visualize calcium transport between cells after a target single cell was stimulated by the developed ultrasonic transducer.

Single-cell engineering has great potential to visualize active molecular events during cell cycle while studying cell–cell interactions and sort intact cells without labeling.^1–7^ While lasers have been used for single-cell-level manipulation due to their short wavelengths, photobleaching and thermal damage to cells may affect cell viability and functional and phenotypical changes after the manipulation.^8,9^ As the fabrication technique of ultrahigh-frequency ultrasonic transducers ranging from 60 MHz to 300 MHz has emerged in the last two decades,^10–12^ the wavelength of ultrasound is comparable to the size of single cells, which allows us to manipulate single cells and micrometer-sized particles with minimal disturbance in deep tissues. Ultrahigh-frequency ultrasound-based imaging improves the spatial resolution to better identify abnormalities in tissue samples. We have developed intravascular ultrasound (IVUS) imaging transducers to improve the resolution for detecting lesion on the lumen wall^12,13^ and single-cell intracellular delivery of various types of macromolecules into cell cytoplasm^5,6^ using ultrahigh-frequency transducers up to 150 MHz. Single-cell acoustic trapping^14^ and cell signaling monitoring using transducers with the center frequency of up to 200 MHz were demonstrated.^3,4,14^

The main and immediate questions using ultrahigh-frequency ultrasonic transducers are the measurements of acoustic radiation force (ARF) at the focus of transducers. Current technology is not mature enough to measure ARF of ultrahigh-frequency ultrasound with the center frequency of over 60 MHz.^15,16^ Many applications using ultrahigh-frequency ultrasound only refer to the input voltages and duty factor without measuring actual ARF at focus. Numerical simulation using commercial packages was used to estimate ARF as an alternative.

Since we engineered immune cells and developed the next generation intracellular delivery technique using ultrasonic transducers, our lab standardized transducer's specification such as aperture diameter, center frequency (*fc*), *fnumber*, and housing sizes to systemically optimize the performance of transducers for these applications and to investigate biological effects of high-frequency ultrasonic transducers to cells.^5,6,17–20^

Here, we quantitatively measured ARF of ultrahigh frequency transducers, which provides a better understanding of the mechanism of high frequency ultrasound-based single-cell manipulation. We estimated ARF of a pushing ultrasonic transducer [1, PUT in Figs. 1(a) and 1(b)] using a model-based approach by mapping measured displacements of a solid sphere in gelatin phantom with theoretical predictions. The theoretical model of the motion of a solid sphere in a viscoelastic medium was developed and experimentally validated in our lab.^21–24^ In our previous study, we developed a theoretical model of a gas bubble and a solid sphere under ARF and measured the mechanical properties of the crystalline lens and the vitreous humor of bovine and porcine eyes using a 3-MHz transducer for pushing and a 25-MHz transducer for tracking.^25–28^ In this study, we used a 130-MHz PUT to displace a solid sphere in a gelatin phantom and a 45-MHz tracking ultrasonic transducer (TUT) to track the displacement of the sphere. If this approach is established to measure the ARF of ultrahigh frequency ultrasonic transducers, this approach will become a gold standard to be widely used in ultrasonic community.

**FIG. 1.**
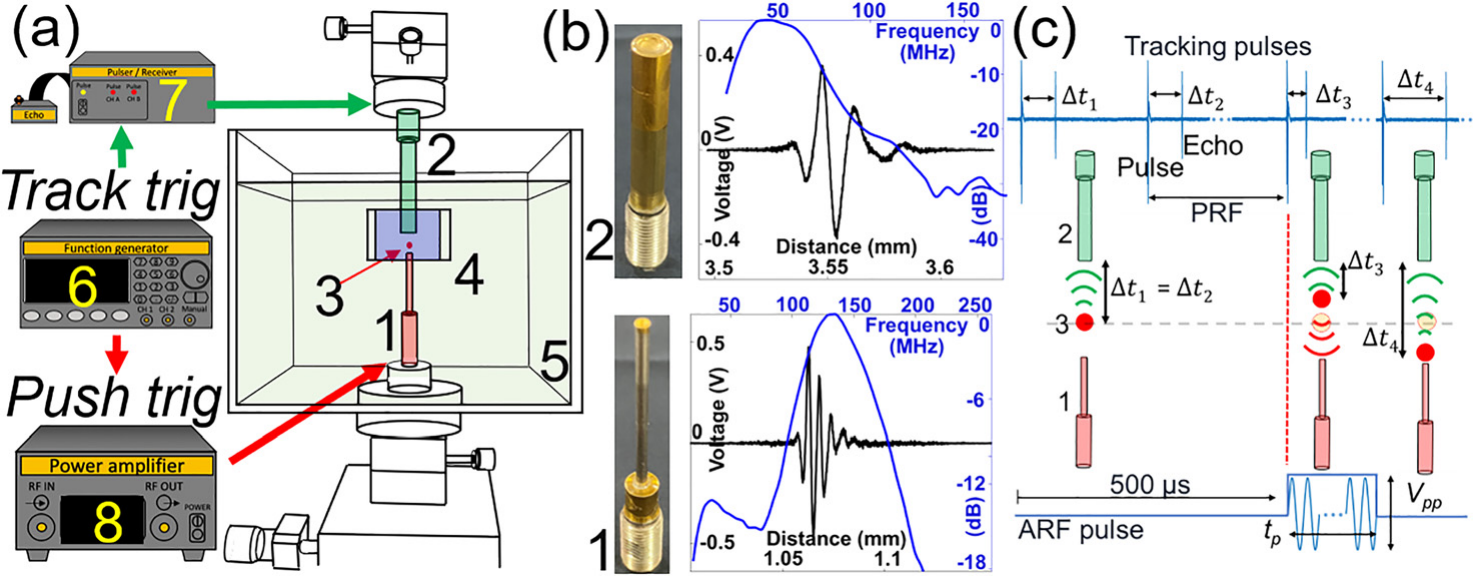
(a) Measurement system of acoustic radiation force (ARF) of a ultrahigh frequency transducer is composed of (1) a pushing transducer (PUT) with (b) the center frequency of 130 MHz, (2) a tracking transducer (TUT) with (b) the center frequency of 45 MHz, (3) a solid sphere, (4) gelatin phantom block, (5) water cuvette, (6) function generator, (7) pulser/receiver, and (8) power amplifier. Red arrows indicate a sequence for pushing triggering to generate ARF in (c), and green arrows indicate a sequence for tracking triggering to generate tracking pulses in (c). (c) When a sphere is moving under ARF from PUT, Δt changes. *V_pp_* is peak-to-peak voltage, and *t_p_* is the pulse duration of ARF pulse. Pulse and echoes were saved for post-processing to reconstruct the displacement of a sphere ball using cross-correlation method.

To develop a system, we fabricated a PUT and TUT using lithium niobate plates (LNB, Boston Piezo-Optics) by following our lab's protocol.^12^ SEM was used to measure the thickness of LNB of the PUT, and it was approximately 10 *μ*m, which matched our designed specification. The measured center frequency (*fc*) of the PUT and TUT was 130 MHz and 45 MHz shown in Fig. 1(b) and Table I.

**TABLE I.** Specification of pushing and imaging ultrasonic transducers.

	*fc* (MHz)	LNB thickness (*μ*m)	Aperture (mm)	*fnumber*	Bandwidth (BW) (%)
TUT	45	50	3	1.16	80
PUT	150	10	1	1	58

We first placed a carbon steel sphere (CS) or a ruby sphere (Ruby) in a 2% gelatin phantom. We measured shear elastic modulus (*μ*) using a Discovery Hybrid HR-20 rheometer (TA instruments). The measured shear elastic modulus of 2% gelatin phantom was 2300 ± 900 Pa from ten specimens. The PUT and TUT were carefully aligned using a goniometer and translation stages with a CS or a Ruby sphere as shown in Fig. 1(a). PUT was positioned in the gelatin phantom block with a sphere to minimize ARF loss due to reflection at water and gelatin phantom boundary. After filling the water cuvette with water, the PUT was connected to a power amplifier [8 in Fig. 1(a)] and the TUT was connected to a pulser/receiver [7 in Fig. 1(a)] and an oscilloscope. A function generator [6 in Fig. 1(a)] controlled tracking and pushing triggering for radio frequency (RF) data acquisition for post-processing to reconstruct the displacement of a sphere under ARF [Fig. 1(c)]. Tracking pulses were emitted from the TUT with a pulse repetition frequency of 100 kHz (PRF), and a pushing pulse was emitted from the PUT with a pulse duration (*t_p_*) and peak-to-peak voltage (*V_pp_*) as shown in Fig. 2(c). Five hundred pulse/echo was saved in a storage for post-processing to visualize the displacement of a sphere. The sampling frequency (*f_s_*) was 10 G_samples_/s, and 50 × 10^6^ samples were saved to track the dynamics of the sphere for 5 ms. The cross correlation speckle tracking method was used to define the displacement of the sphere.^29^

**FIG. 2.**
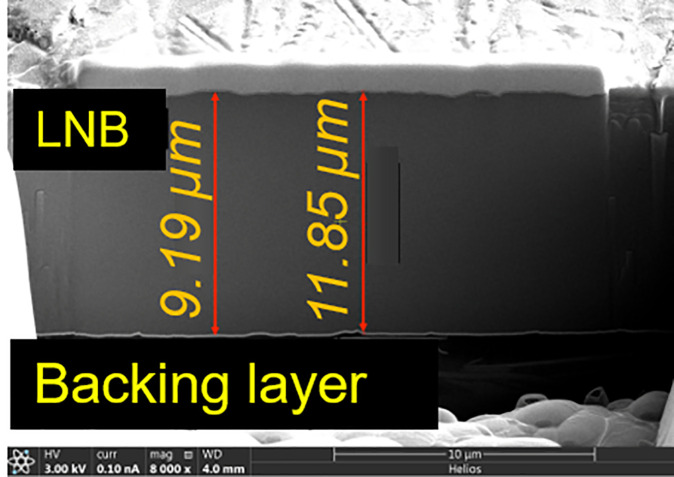
SEM image of LNB and backing layer of TUT.

The previously developed and validated theoretical model formulates the dynamics of a solid sphere in a viscoelastic medium under impulsive ARF. The densities of CS (7850 kg/m^3^) and Ruby (4000 kg/m^3^) were used. A brief summary of a theory is the following. We used transient and impulse-like acoustic pulse to displace a solid sphere in nearly incompressible and elastic medium. Similar to the Stoke's formula for a solid sphere moving in liquid, the displacement of a solid sphere in an elastic medium can be found using the balance equation for stress tensor and strain tensor.^23,30^ The governing equation of motion for a solid sphere in an elastic medium in a frequency domain is^22,23,26^

$$-\rho \omega^{2}u=\mu \nabla^{2}u-\nabla p,$$ (1)

where *ρ* is the medium density, p is the pressure in the medium in the frequency domain, ω is the oscillation frequency under excitation (EX) frequency, u is the displacement vector, and *μ* and *η* are the shear elastic modulus and shear viscosity, respectively.

The general solution of Eq. (1) is

$$f\left(r\right)=\frac{\tilde{a}e^{\mathrm{ikr}}}{\mathrm{ikr}}-\frac{\tilde{b}}{r},$$ (2)

where $\tilde{a}$ and $\tilde{b}$ are complex constants to be determined by the boundary conditions. $k=\frac{\omega }{c_{t}}$ and $c_{t}=\sqrt{\frac{\mu }{\rho }}$, r is the radial direction in the polar coordinate system.

Displacement components of u are

$$u_{r}=-\frac{2}{r^{2}}\left[\tilde{a}e^{\mathrm{ikr}}\left(1-\frac{1}{\mathrm{ikr}}\right)+\frac{\tilde{b}}{r}\right]\text{cos}\;\theta ,$$ (3)

$$u_{\theta }=-\frac{1}{r^{2}}\left[\tilde{a}e^{\mathrm{ikr}}\left(1-\mathrm{ikr}-\frac{1}{\mathrm{ikr}}\right)+\frac{\tilde{b}}{r}\right]\text{sin}\;\theta .$$ (4)

Boundary conditions at the surface of a solid sphere (*r* = *R*),

$$u_{r}\left(R,\theta \right)=u_{0}\;\text{cos}\;\theta ,$$ (5)

$$u_{\theta }\left(R,\theta \right)={-u}_{0}\;\text{sin}\;\theta ,$$ (6)

were used to find constants $\tilde{a}$ and $\tilde{b}$ as

$$\tilde{a}=-\frac{3Ru_{w}}{2ik}e^{\mathrm{ikR}},$$ (7)

$$\tilde{b}=-\frac{R^{3}u_{w}}{2}\left(1-\frac{3}{\mathrm{ikR}}-\frac{3}{k^{2}R^{2}}\right),$$ (8)

where θ is an angle between the radial direction and displacement of a sphere, $u_{0}$ is the magnitude of u, and $u_{w}$ is the spectral component of a displacement, which will be inverse Fourier transformed at the end to find the actual displacement of a solid sphere in the time domain.

Displaced solid spheres are experiencing forces similar to the drag force in response to the medium on the surface of a solid sphere,

$$F=2\pi R^{2}\int_{0}^{\pi }(-p\;\text{cos}\;\theta +\sigma_{rr}\;\text{cos}\;\theta {-\sigma }_{r\theta }\;\text{sin}\;\theta )\text{sin}\;\theta d\theta .$$ (9)

The pressure gradient in Eq. (1) and Eqs. (7) and (8) are used to find an expression for pressure:^23^

$$p=p_{0}-\frac{\mu k^{2}R^{3}u_{w}}{2r^{2}}\left(1-\frac{3}{\mathrm{ikR}}-\frac{3}{k^{2}R^{2}}\right)\text{cos}\;\theta .$$ (10)

Stress components are

$$\sigma_{r\theta }=2\mu u_{r\theta }=\mu \left(\frac{\partial u_{\theta }}{\partial r}-\frac{u_{\theta }}{r}+\frac{1}{r}\frac{\partial u_{r}}{\partial \theta }\right)=\frac{3\mu u_{w}}{2R}\left(1-\mathrm{ikR}\right)\text{sin}\;\theta ,$$ (11)

$$\sigma_{rr}=0.$$ (12)

Equations (10)–(12) are substituted in Eq. (9) to find the spectral component of force acting on a solid sphere from elastic medium

$$F_{w}=-6\pi \mu Ru_{w}\left(1-\mathrm{ikR}-\frac{k^{2}R^{2}}{9}\right).$$ (13)

To consider viscoelastic medium, μ and k are changed to (μ−iωη) and $k=\frac{\omega }{\left(c_{t}\sqrt{1-\frac{i\omega \eta }{\mu }}\right)}$, where η is a shear viscosity coefficient.

To couple the displacement of a solid sphere and externally applied force to a sphere in frequency domain (${F_{w}}^{\left(\mathrm{ext}\right)}$) using Newton's second law

$${F_{w}}^{\left(\mathrm{ext}\right)}+F_{w}=-M\omega^{2}u_{w},$$ (14)

$${F_{w}}^{\left(\mathrm{ext}\right)}=6\pi (\mu -i\omega \eta )Ru_{w}\left(1-\mathrm{ikR}-\frac{k^{2}R^{2}}{9}\right)-M\omega^{2}u_{w},$$ (15)

where M is a mass of a solid sphere. The spectral component of the displacement of a solid sphere is obtained from Eq. (15)

$$u_{w}=\frac{{F_{w}}^{\left(\mathrm{ext}\right)}}{6\pi (\mu -i\omega \eta )R\left(1-\mathrm{ikR}-\frac{k^{2}R^{2}}{9}\right)-M\omega^{2}}.$$ (16)

$u_{w}$ can be determined under impulsive and transient external force defined as follows [Fig. 1(c)]:

$$F^{\left(\mathrm{ext}\right)}=\left\{\begin{array}{ll} F_{0\;} & 0\leq t\leq t_{p},\\ 0 & t\geq t_{p}, \end{array}\right.$$ (17)

where $F_{0}$ is ARF to be measured and $t_{p}$ is the duration of acoustic radiation pulse [Fig. 1(c)]. In the frequency domain, Eq. (17) becomes

$${F_{w}}^{\left(\mathrm{ext}\right)}=-\frac{iF_{0}}{\omega }\left(e^{i{\omega t}_{p}}-1\right).$$ (18)

Once $u_{w}$ is found using Eqs. (16) and (18), an inverse Fourier transform {$\mathrm{inverse}\;\mathrm{Fourier}\;\left[f(\omega )\right]=\frac{1}{2\pi }\int_{-\infty }^{\infty }f\left(\omega \right)e^{-i\omega t}d\omega \}$ is performed to determine $\tilde{a}$ and $\tilde{b}$ in Eqs. (7) and (8). Then, using Eqs. (3) and (4), $u_{r}$ and $u_{\theta }$ can be found to determine the displacement of a solid sphere under ARF. Since we consider the displacement of a sphere along the ultrasound propagation, *θ* is set to be zero. Therefore, $u_{r}$ represents the displacement of a sphere under consideration.

We first used a 150-*μ*m radius CS sphere to investigate the feasibility of mapping between measurements and theoretical calculations of the dynamics of a solid sphere under ARF generated by a 130-MHz transducer. To estimate ARF ($F_{0}$), $t_{p}$ was fixed to 20 *μ*s and *V_pp_* was increased from 30  to 90 mV. These values were chosen from other publications of our lab's^3,5,6^ for the intracellular delivery of macromolecules and acoustic single-cell stimulation. Because the gain of the power amplifier is 50 dB, 30 and 90 mV represent 9  to 28 V. Measured displacements of a CS sphere are plotted in Fig. 3(a), and the theoretical calculation to match with the measured displacements is shown in Fig. 3(b). After matching between measurements and theory, $F_{0}$ was estimated to be 60 , 40 , 24 , and 9 *μ*N for 90 , 70 , 50 , and 30 mV, respectively. To confirm estimated $F_{0}$, we fixed 70 mV and changed $t_{p}$ as 1, 10, 20, and 30 *μ*s. If $F_{0}$ of 40 *μ*N was correctly estimated, theory and measurement using different $t_{p}$ values should be matched. As shown in Figs. 3(c) and 3(d), measured displacements and theoretical calculations match for varying $t_{p}$ values with $F_{0}$ of 40 *μ*N. Therefore, the measured ARF of *V_pp_* of 90 , 70 , 50 , and 30 mV with fixed 20 *μ*s was confirmed as 60 , 40 , 24 , and 9 *μ*N, respectively.

**FIG. 3.**
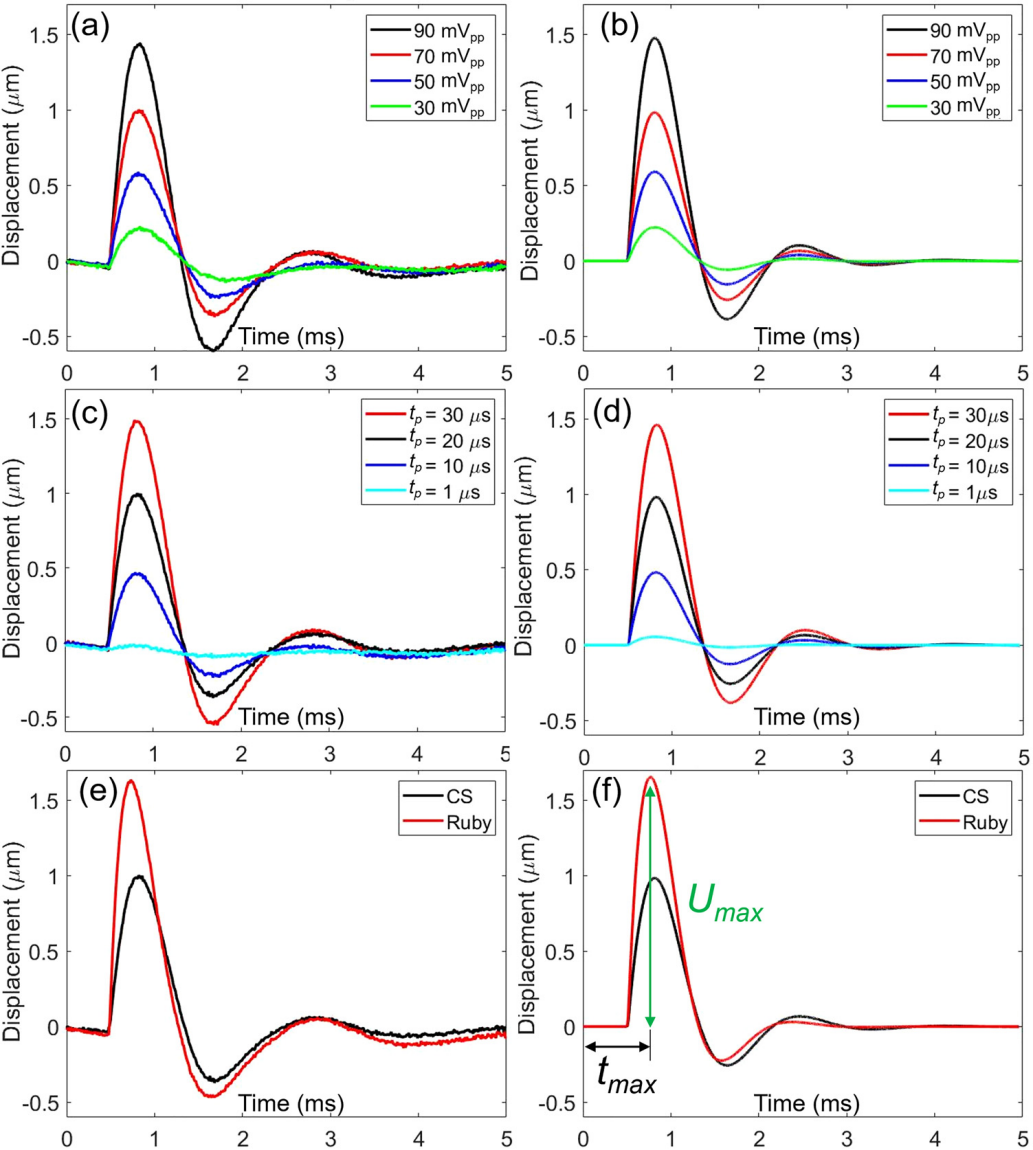
(a) Measured displacements and (b) theoretical calculations of a carbon steel sphere (CS) depending on *V_pp_.* Pulse duration ($t_{p}$) is 20 *μ*s. (c) and (d) To confirm mapping between measurements and a theory, $F_{0}$ and *V_pp_* are fixed while $t_{p}\;$ is changed from 1 to 30 *μ*s. (e) and (f) To confirm mapping between measurements and a theory, a ruby sphere (Ruby) and CS were compared. *U_max_* and *t_max_* represent maximum displacement of a sphere and time to reach *U_max_*.

As a second confirmation, we changed the material of spheres. We used a 150-*μ*m-radius Ruby sphere and compared the displacements of a Ruby sphere with a CS sphere, when *Vpp* is 70 mV and $t_{p}\;$ is 20 *μ*s. Considering the density of Ruby (4000 kg/m^3^) and CS (7850 kg/m^3^), time to reach maximum displacement (*t_max_*) was decreased and maximum displacement (*U_max_*) was increased as shown in Figs. 3(e) and 3(f). Under the same ARF, the lighter Ruby sphere travels longer distance within a short time.

Because we confirmed this model-based ARF measurements by varying *V_pp_*, $t_{p}$, and the materials of a sphere, we measured ARF and impulse by comparing *U_max_* of a CS and Ruby spheres within a design space of *V_pp_* of 30 , 50 , 70 , and 90 mV and $t_{p}$ of 5, 10, 15, 20, and 30 *μ*s. We selected *V_pp_* and $t_{p}$, which have been frequently used for intracellular delivery of macromolecules, acoustic tweezers, and single cell stimulations using ultrahigh frequency ultrasound. The PUT, TUT, and a sphere were co-aligned in gelatin phantom not to cause any reflection and refraction, which may occur when ultrasound waves are traveling across water and gelatin boundaries. Measured $F_{0}$ are 9.9 ± 1.4 , 26.5 ± 4.2 , 42.4 ± 3.0 , and 65.1 ± 5.8 *μ*N for 30 , 50 , 70 , and 90 mV, respectively, as shown in Fig. 4(a). We can conclude here that the applied ARF to cells during intracellular delivery and single cell stimulation was in the range of several tens of *μ*N.

**FIG. 4.**
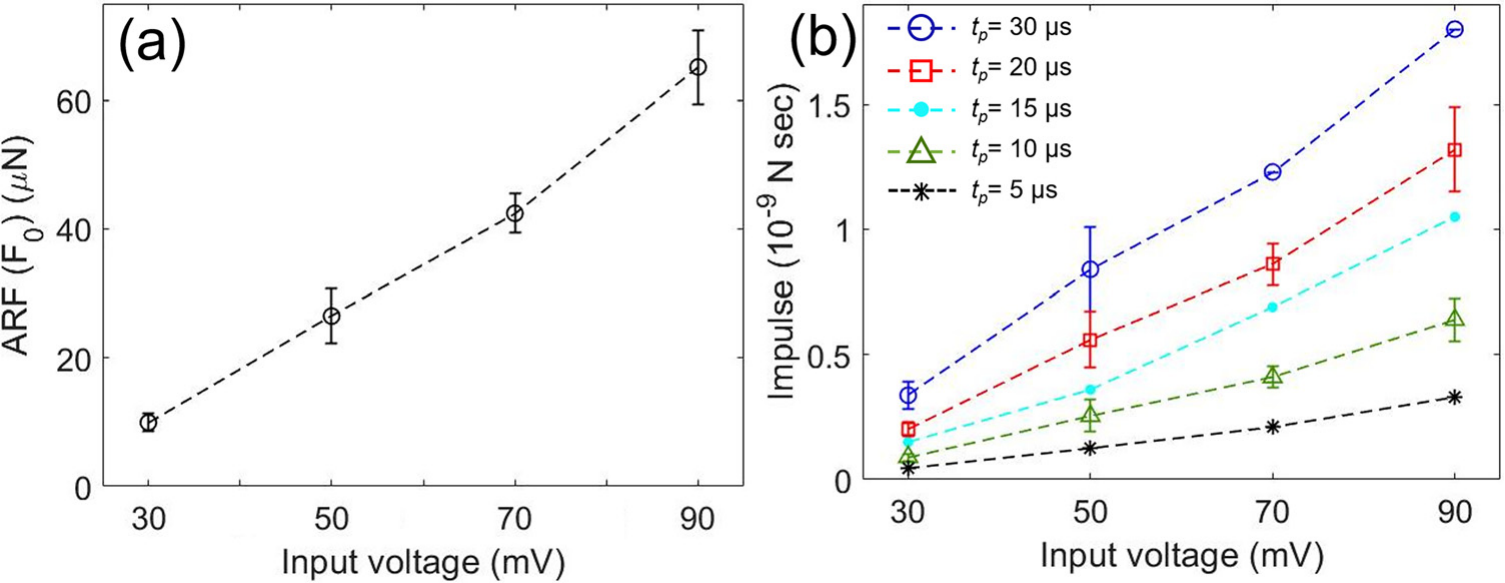
(a) Measured ARF (*F*_0_) of 130 MHz ultrahigh-frequency transducer is ranging from 10  to 65 *μ*N. (b) Impulse (*F*_0_·*t_p_*) is estimated to analyze the biophysical influence of high-frequency ultrasound to cells depending on *t_p_*.

To include $t_{p}$ as a means of a biophysical effect of high frequency ultrasound to cells, impulse was estimated as shown in Fig. 4(b). Briefly, the area under ARF ($F_{0}$) and time curve represent momentum changes in time. Momentum changes during $t_{p}$ are impulse, which can be used to measure the biophysical influence on cells of high frequency ultrasound.

We used the PUT to demonstrate single cell stimulation and investigate calcium transport between the cells. An integrated system was used to stimulate a target cell, while capturing fluorescence resonance energy transfer (FRET) changes of the target cell and a neighboring cell to visualize calcium transport between two cells [Fig. 5(a)]. The 3D stage was computer-controlled with a sub-micrometer precision, where the transducer was attached for focusing and stimulation of the target single cell. Foci of the microscope and the transducer were coaligned for FRET-based live cell imaging. To prevent standing waves between a PUT aperture and the bottom of a cell culture dish, PUT was positioned at 45° with respect to cell culture dish [Fig. 5(a)]. Particularly, standing waves induce pressure doubling under the rigid boundary condition.

**FIG. 5.**
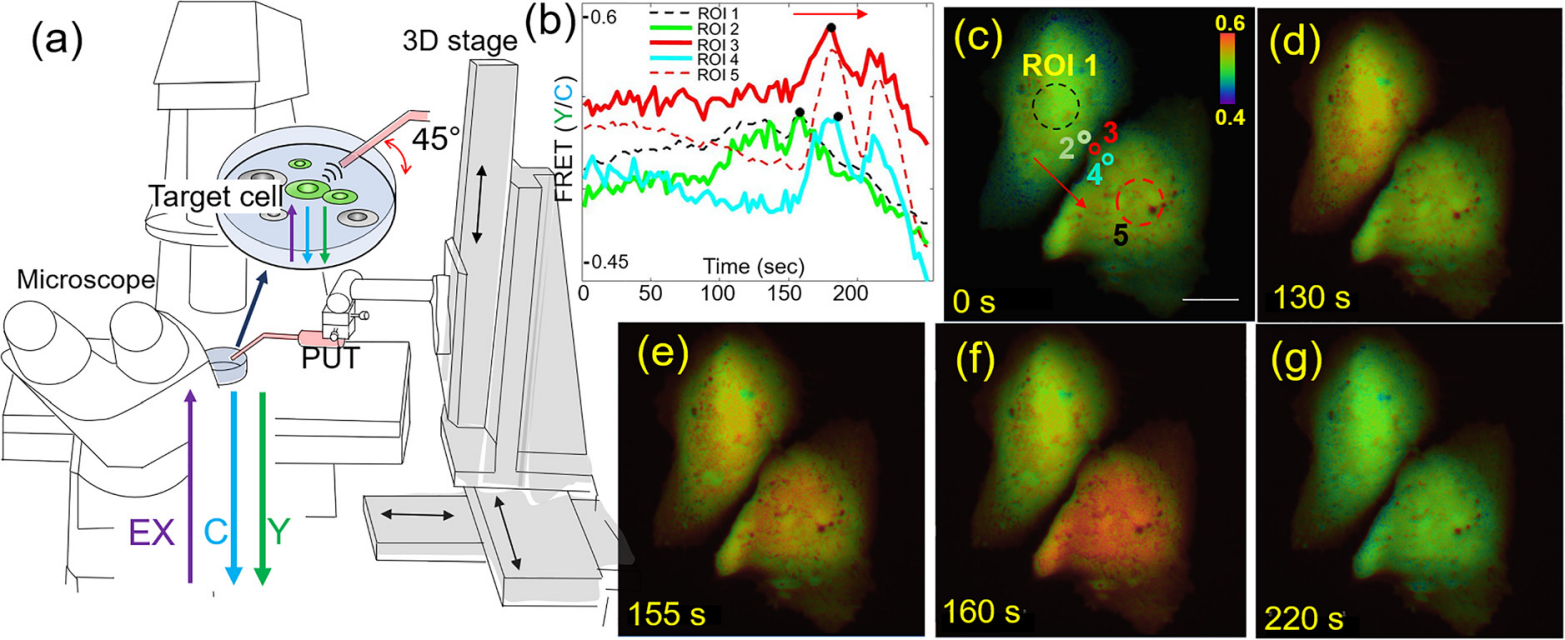
(a) PUT with 130 MHz center frequency stimulates a target cell expressing FRET biosensor to detect calcium transport between cells. PUT position is controlled by 3D stage. Microscope was used to image FRET changes of target cells after the stimulation using the intensity ratio between ECFP (C) and YPet (Y). (b) and (c) Time course changes of FRET of ROIs 1–5. ROI 1 is in the middle of the target cell and ROI 5 is in the middle of a neighboring cell. ROIs 2–4 are sequentially located from the stimulated target cell to the neighboring cell. Black dots indicate maximum FRET of ROIs 2–4. FRET changes of ROIs 2–4 indicate calcium transport as indicated red arrows and black dots on the FRET plot. A scale bar indicates 20 *μ*m. The color scale bar in panel (c) represents the range of FRET ratio with purple and red colors, indicating low and high levels of calcium concentration, respectively. (d)–(g) FRET images of two cells at the designated time.

Ultrasound pulses with *V_pp_* of 70 mV and $t_{p}\;$ of 5 *μ*s were applied to the region of interest 1 (ROI 1) shown in Fig. 5(c) for 1 min between 90 s and 150 s with a PRF of 1 kHz. From ARF measurements in Fig. 4(a), 42.4 ± 3.0 *μ*N ARF was applied every 1 ms for 1 min. FRET images were collected with the Ti2 Nikon fluorescence microscope and a cooled charge-coupled device (CCD) camera using the Nikon NIS-Elements AR software. Nikon NIS-Elements AR was used to calculate the pixel-by-pixel ratio images of FRET-YPet over Enhanced cyan fluorescent protein (ECFP) after subtracting the basal fluorescence level. In other words, FRET is the ratio between the intensity of the YPet signal [Y in Fig. 5(a)] and ECFP signal [C in Fig. 5(a)], represented by a simple equation (FRET = Y/C) in Fig. 5(a). A high FRET value of 0.6, in this case, represents an increased intracellular calcium concentration, and a low FRET value of 0.4 indicates a decreased and basal calcium level in cytoplasm of cells. Microscope filter setting for ECFP images was excitation (EX) 440/20 nm and emission (EM) 480/40 nm. FRET-YPet image filter setting was EX 440/20 nm and EM 535/30 nm. Calcium transport between two cells was clearly visualized due to the time shift between maximum FRET in ROIs 2–3 [black dots in Fig. 5(b) and red arrows in Figs. 5(b) and 5(c)]. Figure 5(c)–5(g) represents FRET images at 0, 130, 155, 160, and 220 s, respectively. First FRET increases only within the stimulated cell as shown in Fig. 5(d). Then, FRET increases in both the cells as shown in Fig. 5(e). Quantitatively, the first FRET peak of ROI 1 is at 155 s and the first FRET peak of ROI 5 is at 182 s. Peak FRET of ROIs 2–4 shifts from 159, 181, and 184 s, respectively. These results confirm calcium transport between two cells starting from the stimulated cell.

We developed an approach to measure ARF of ultrahigh-frequency ultrasonic transducers using the theoretical model of the dynamics of a sphere in a gelatin phantom. The impulse due to ARF was estimated to include the influence of $t_{p}$. To estimate ARF at the focus of a 130 MHz transducer, we matched measured *U_max_* with theoretical calculations. We selected appropriate ranges of *V_pp_* and $t_{p}$ for single-cell applications, and estimated ARFs were in the range of tens of *μ*N. To increase the accuracy of the proposed approach in this paper, the absorption and boundary conditions of different targets should be considered. In this paper, we used a solid sphere to measure ARF of the ultrahigh-frequency ultrasound. ARF for cell applications needs some corrections. The boundary condition between the gelatin medium and solid sphere can be approximated as a rigid boundary condition, where pressure doubling, a phase change, and nearly perfect reflection occur. On the other hand, the boundary condition for cells may be considered as a pressure release boundary condition, where no pressure doubling, no phase change, and less reflection occur. Due to these reasons, the measured displacements may be smaller than what we observed in this paper. To further fine-tune the proposed method, point targets with impedance similar to cells may be used for future study. FRET live cell imaging was performed to visualize calcium transport between cells after a target single cell was stimulated by the developed ultrasonic transducer. We conclude that the calcium ion may be intracellularly delivered by acoustic radiation force in the range of *μ*N. Because the focal diameter of 130 MHz is approximately 10 *μ*m, the estimated pressure was 0.54 MPa using ARF of 42.4 *μ*N and used to intracellularly deliver calcium ions. This study may establish a gold standard to estimate ARF of ultrahigh-frequency transducers for single-cell applications.

## Data Availability

The data that support the findings of this study are available from the corresponding author upon reasonable request.
